# Hypoxia-induced immunosuppression in uveal melanoma is mediated by CD63^+^ exosomes delivering lactate to reprogram immune cells

**DOI:** 10.3389/fmolb.2025.1743009

**Published:** 2026-01-07

**Authors:** Lei Dong, Suilian Zheng, Yixia Feng

**Affiliations:** 1 Department of Ophthalmology, The Third Affiliated Hospital of Wenzhou Medical University, Ruian, Zhejiang, China; 2 Department of Ophthalmology, The Second Affiliated Hospital & Yuying Children’s Hospital of Wenzhou Medical University, Wenzhou, Zhejiang, China

**Keywords:** exosomes, hypoxia, immunosuppression, lactate, uveal melanoma

## Abstract

Uveal melanoma (UM) is characterized by profound immunosuppression, resistance to immunotherapy, and significant hypoxia. This study investigates the role of hypoxia in mediating metabolic crosstalk with immune cells via CD63-enriched exosomes. Single-cell transcriptomic analysis identified a CD63-high tumor subpopulation in UM associated with lactate metabolism and vesicle transport. Under hypoxic conditions (1% O_2_ vs. 21% O_2_ normoxia), UMT2 cells exhibited upregulation of CD63 expression, increased exosome secretion, and elevated exosomal lactate levels. In co-culture assays, these hypoxic exosomes promoted macrophage M2 polarization, as indicated by increased CD206^+^ expression and elevated Extracellular Acidification Rate/Oxygen Consumption Rate (ECAR/OCR) ratios in macrophages and induced CD8^+^ T cell exhaustion, as evidenced by higher PD-1^+^TIM-3^+^ expression, and promoted the secretion of immunosuppressive cytokines such as TGF-β and IL-10. Importantly, these effects, which were driven by exosomal lactate transfer leading to macrophage metabolic reprogramming, were abolished upon CD63 knockdown using siRNA. Mechanistically, CD63 facilitates a hypoxia-induced exosomal lactate shuttle. We conclude that CD63-mediated transfer of hypoxic exosomal lactate establishes a critically immunosuppressive microenvironment in UM. Targeting the hypoxia/CD63/exosomal lactate axis may represent a promising novel therapeutic strategy to restore anti-tumor immunity in UM.

## Graphical Abstract

**Figure:**
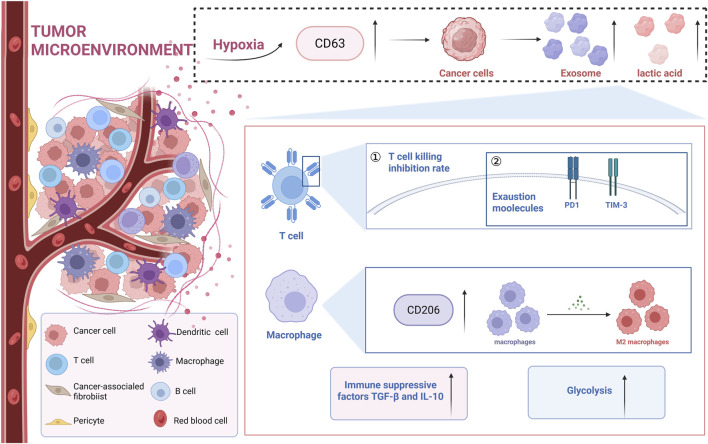
Schematic depicting hypoxic myeloma cells secrete CD63^+^ exosomes to deliver lactate and promote immunosuppression. Graphical Abstract was created by biorender and licensed to be published.

## 1 Introduction

Uveal melanoma (UM) is the most common intraocular malignant tumor in adults, presenting significant challenges due to its high metastatic potential and limited treatment options (Chan et al., 2024; Păsărică et al., 2024; Ziogas et al., 2025). Despite progress in local tumor control, up to 50% of patients are still experiencing metastasis, mainly liver, with a median survival period of only 4–5 months following metastasis. In cases with metastasis, the 2- and 5-year survival rates are approximately 10% and less than 1%, respectively (Barbagallo et al., 2023; Mirzayev et al., 2024; Zameer et al., 2024; Yamashita et al., 2025). Hypoxia, a hallmark of solid tumors, is especially prominent in UM. It drives tumor aggressiveness by promoting angiogenesis, reprogramming metabolism, and facilitating immune evasion (Chen et al., 2022; Zhao et al., 2022; Fan et al., 2023; Păsărică et al., 2023). However, the molecular pathways linking hypoxia to immune suppression in UM remain incompletely understood. Despite the success of immunotherapy in cutaneous melanoma, UM remains largely refractory to immune checkpoint blockade, underscoring the urgent need to understand its unique immunosuppressive landscape, of which hypoxia is a cardinal feature.

Exosomes, which are nanoscale extracellular vesicles measuring 30–150 nm and derived from multivesicular bodies, serve as critical mediators of intercellular communication within the tumor microenvironment (Huang et al., 2022; Altıntaş and Saylan, 2023; Li et al., 2025). Under hypoxic conditions, tumor-derived exosomes exhibit altered cargo composition, including metabolites, proteins, and nucleic acids. This altered cargo transmits the hypoxia tolerance of tumor cells to immune cells, resulting in adaptive changes in their phenotypes and functions (Beaumont et al., 2022; Shao et al., 2022; Tang et al., 2022). Lactate, a major product of tumor cell glycolysis, has emerged as a key metabolic mediator in cancer-immunity interactions. Increased levels of lactate within the tumor microenvironment facilitate the transformation of macrophages into an immunosuppressive M2 phenotype, inhibit T cell function, and correlate with poor clinical outcomes across various cancers (American Association for Cancer Research, 2022; Tian et al., 2022). However, the mechanisms by which lactate is shuttled between tumor cells and immune cells under hypoxic conditions, as well as the role of exosomal carriers in this process, remain unclear.

CD63, a protein that is highly expressed on the membranes of exosomes, plays a crucial role in regulating exosome biogenesis, cargo sorting, and cellular uptake (Su et al., 2023; Palmulli et al., 2024). Recent studies have demonstrated that hypoxia is associated with the upregulation of CD63 expression (Wu et al., 2022; Wang et al., 2024). The literature indicates that lactate in tumors can promote the development and function of CD63-immunomodulatory dendritic cells through an SREBP2-dependent mechanism (Plebanek et al., 2024). However, it remains entirely unknown whether CD63 mediates lactate shuttling in hypoxic UM cells and contributes to the reprogramming of the immune microenvironment.

In this study, we tested the hypothesis that hypoxia promotes increased CD63 expression in myeloma cells, which leads to enhanced exosome secretion and elevated lactate levels, leads to the polarization of M2 macrophages and the exhaustion of T cells by combining molecular, cellular, and immunological strategies, we aimed to elucidate this novel immunosuppressive mechanism involving lactate shuttling under hypoxia-induced CD63 upregulation and to identify potential therapeutic targets for reversing this process.

## 2 Methods

### 2.1 Single-cell sequencing

Data pertinent to the study were obtained from the Gene Expression Omnibus (https://www.ncbi.nlm.nih.gov/geo/info/datasets.html), a database of gene expression information curated by the National Center for Biotechnology Information. The analysis of gene expression profiles was conducted using the Seurat package. Following this, genes exhibiting low expression levels (%mt < 20) were excluded from the analysis. The dataset was subjected to normalization, mean-centering, principal component analysis, and harmonization. To visualize the spatial relationships among clusters, Uniform Manifold Approximation and Projection (UMAP) analysis was applied.

### 2.2 GO and KEGG analysis

The Gene Ontology Federation has established a significant database known as GO. This resource provides a thorough framework for defining the biological processes, cellular components, and molecular functions linked to a diverse range of genes. On the other hand, the KEGG serves as a comprehensive database that consolidates data on biological pathways, genomes, chemical substances, drugs, and diseases, being widely used for the analysis of metabolic and functional pathways. To explore the connections between functions and pathways, we analyzed the essential roles of differentially expressed genes (DEGs) via GO and pathway analyses (Xu et al., 2024). Enrichment analyses for DEGs were performed with clusterProfiler (version 4.0), the threshold of the P value is below 0.05, indicating statistical significance. Furthermore, to visualize the outcomes, ClueGo, a plugin for Cytoscape, was employed to develop the GO and KEGG networks.

### 2.3 Cell culture

Human melanoma cell lines (UMT2), THP-1 macrophages derived from humans (ATCC TIB-202), and CD8^+^ T cells were sourced from the Type Culture Collection of the Chinese Academy of Sciences located in Shanghai, China. The cell lines were maintained in DMEM. All the cell lines underwent Short Tandem Repeat DNA profiling to confirm their identity. The UMT2 cell line used in this study harbors a GNA11 mutation (Suesskind et al., 2013).

### 2.4 Isolation of exosomes

To isolate exosomes, the supernatant obtained from melanoma cells that were cultured in DMEM supplemented with exosome-depleted fetal bovine serum (FBS) for 48 h was used. The supernatant was subjected to a series of centrifugation steps at 4 °C: initially at 300 × g for 10 min, then at 3,000 × g for 30 min, and finally at 10,000 × g for 30 min. The resulting supernatant was filtered through a 0.22-μm PES filter (Millipore). Subsequently, ultracentrifugation was performed at 100,000 × g for 70 min at 4 °C using an Optima XE-90 Supercentrifuge (Beckman, Germany). The pellet was washed with 30 mL of 1× PBS (pH 7.4) and then subjected to a second ultracentrifugation at 100,000 × g for 70 min at 4 °C to obtain the purified exosomes.

### 2.5 Nanoparticle tracking analysis (NTA)

NTA was implemented following the guidelines provided by the manufacturer using the Malvern Zetasizer Nano ZS-90. The exosomes were prepared by diluting them in PBS. Dynamic light scattering with the Malvern Zetasizer Nano ZS-90 was utilized to ascertain the average particle size and the distribution of sizes.

### 2.6 Transmission electron microscopy (TEM) for exosome morphology

Isolated exosomes were prepared for TEM imaging to confirm their characteristic morphology and purity. Briefly, 10 μL of purified exosome suspension was applied onto a carbon-coated copper grid and allowed to adsorb for 5 min at room temperature. Excess liquid was carefully removed using filter paper. The sample was then negatively stained with 10 μL of 2% (w/v) uranyl acetate solution for 1 min. After removing the stain and air-drying for 10 min, the grid was examined under a transmission electron microscope (Hitachi HT7800, Japan) operated at an acceleration voltage of 80 kV. Images were captured at various magnifications using a digital imaging system.

### 2.7 Protein extraction and western blotting

Cells and exosomes were lysed using RIPA buffer supplemented with protease and phosphatase inhibitors. The protein concentration was determined using a BCA protein assay kit. For SDS-PAGE analysis, 10–30 μg of protein per sample were loaded onto gels and subsequently transferred onto PVDF membranes. After transfer, the membranes were washed with 1× TBST and blocked with 5% nonfat milk in 1× TBST for 2 h at room temperature. The membranes were then incubated overnight at 4 °C with the following primary antibodies: anti-CD63 (Proteintech, 25682-1-AP; 1:1000), anti-Alix (Proteintech, 12422-1-AP; 1:5000), anti-TSG101 (Proteintech, 28283-1-AP; 1:1000), and anti-β-actin (Proteintech, 66009-1-Ig; 1:5000). Following primary antibody incubation, membranes were washed three times with TBST and incubated with an HRP-conjugated goat anti-rabbit or anti-mouse secondary antibody (Proteintech, 1:5000) for 2 h at room temperature. After three additional TBST washes, protein bands were visualized using an ECL detection reagent and captured with a chemiluminescence imaging system.

### 2.8 Quantitative PCR

RNA was extracted from cells with the RNeasy Mini Kit, followed by qPCR conducted with Superscript IV Reverse Transcriptase. The 2^−ΔΔCT^ algorithm was utilized to ascertain gene-fold changes, using β-Actin as a reference gene to standardize the 2^−ΔΔCT^ calculations. The specific sequences of the primers for CD63 and β-actin are presented in Table 1.

**TABLE 1:** The specific sequence of different primers.

Gene	Sequence
CD63	Forward: 5′-CCCAAGCTTGCCACCATGGCGGTGGAAGGAGGAATGAAATG-3′ Reverse: 5′-CCGCTCGAGCATCACCTCGTAGCCACTTCTGATAC-3′
β-Actin	Forward: 5′-TGACGTGGACATCCGCAAAG-3′ Reverse: 5′-CTGGAAGGTGGACAGCGAGG-3′

### 2.9 Statistical analysis

The results are presented as mean ± S.E.M. Comparisons of data were performed using one-way ANOVA, two-way ANOVA, and the Student’s t-test, establishing a significance level at p < 0.05. All statistical analyses were performed with GraphPad Prism version 9.0 (La Jolla, CA, United States).

## 3 Results

### 3.1 Single-cell transcriptome analysis of the distribution of CD63 and lactic acid

**FIGURE 1:**
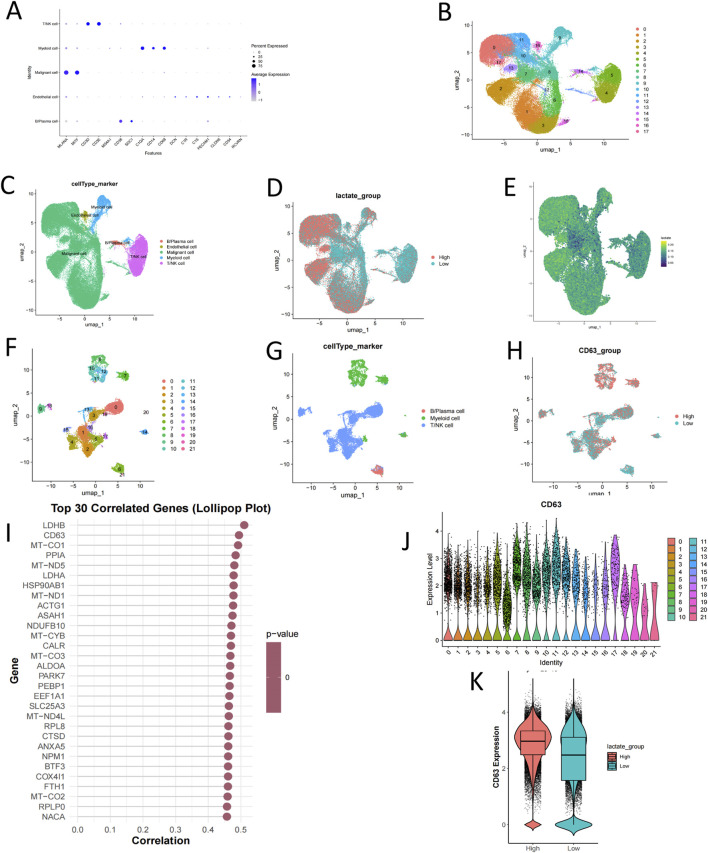
Single-cell transcriptome analysis of the distribution of CD63 and lactic acid in the tumor microenvironment. **(A)** Visualization of gene expression. **(B)** UMAP plot illustrating all cells categorized into 17 clusters, with distinct clusters represented by different colors. **(C)** UMAP-based visualization of cell types. **(D,E)** UMAP plots showcasing the expression of lactate metabolism-related genes across various cell types. **(F)** UMAP plot depicting all cells classified into 21 clusters, with unique colors denoting different clusters. **(G)** UMAP-based visualization of cell types. **(H)** UMAP plot displaying CD63 expression among various cell types. **(I)** Plot of the top 30 genes. **(J)** Results of differential expression analysis for CD63 across inter-group differential cells. **(K)** Correlation analysis between lactate and CD63 expression. Statistical analyses were performed using R (v4.1.1). Kruskal–Wallis rank-sum test was used by ggsingif packages (0.6.3) in R.

Single-cell sequencing analysis revealed significantly elevated expression of lactate metabolism-related genes in the malignant cell clusters of UM tumor cells. High-lactate regions were found to completely overlap with malignant cell clusters, whereas low-lactate regions were predominantly located within the normal cell clusters surrounding the tumor (Figures 1A–E). Furthermore, CD63 exhibited significantly higher expression levels in the malignant cell clusters of immune cells and demonstrated a strong correlation with glycolytic genes, such as LDHA and LDHB, in tumor cells (Figures 1F–J). Additional analysis indicated that increased CD63 levels were associated with elevated lactate concentrations (Figure 1K).

### 3.2 Association of CD63 high expression with vesicle transport

**FIGURE 2:**
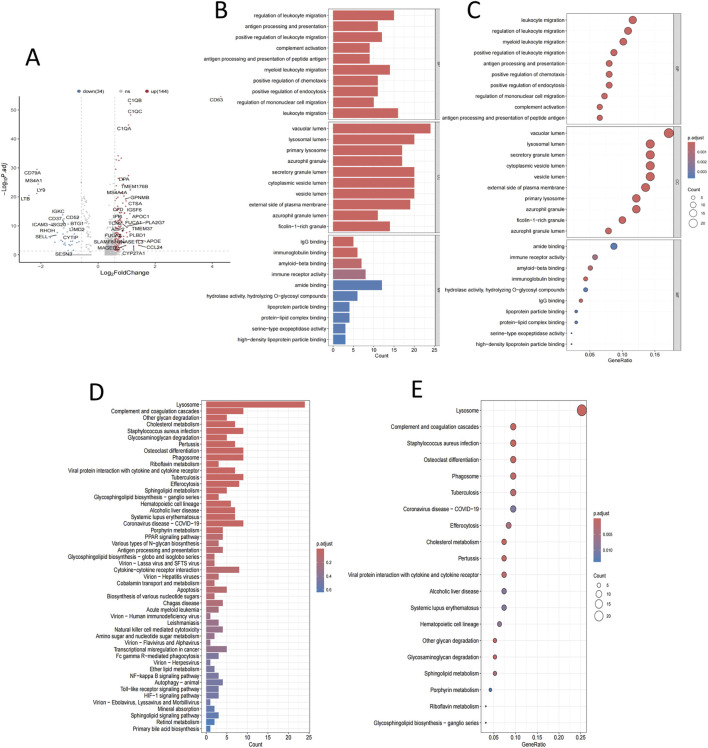
Association of CD63 high expression with vesicle transport. **(A)** Gene expression volcano plot. **(B,C)** GO enrichment analysis. **(D,E)** KEGG enrichment analysis.

Differentially expressed genes were identified using volcano plot filtering. A total of 178 differentially expressed genes were detected, of which 144 were upregulated, including vesicle-related genes such as C1QB, C1QC, C1QA, and LIPA, along with CD63. In contrast, 34 genes were found to be downregulated (Figure 2A). GO enrichment analysis revealed that genes associated with CD63 are significantly enriched in cellular components, including ‘vesicle membrane’ and ‘extracellular exosome’, as well as in biological processes such as ‘vesicle-mediated transport’ and ‘exosome secretion.’ This finding underscores its strong association with vesicular transport functions (Figures 2B,C). Furthermore, KEGG pathway analysis confirmed the involvement of CD63 in vesicle transport-related pathways, including endocytosis, lysosomal pathways, and extracellular matrix receptor interactions (Figures 2D,E).

### 3.3 Hypoxia upregulates CD63 expression

**FIGURE 3:**
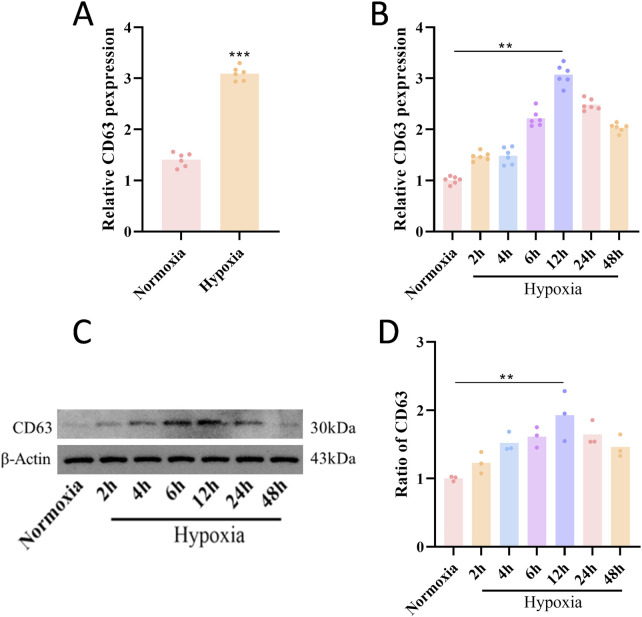
Hypoxia upregulates CD63 expression. **(A)** Expression of CD63 under normoxic and hypoxic conditions (n = 3). **(B)** PCR detects the expression of CD63 under different levels of hypoxia (n = 6). **(C,D)** WB detects the expression of CD63 under different levels of hypoxia (n = 3). The data are presented as mean ± SD. **P < 0.01, ***P < 0.001.

To investigate the regulatory mechanism of hypoxia on CD63, we first subjected the human melanoma cell line UMT2 cells to hypoxic treatment (1% O_2_, 12 h). qPCR analysis demonstrated that CD63 mRNA levels in UMT2 cells were significantly elevated under hypoxic conditions compared to the normoxic group (Figure 3A). Time-gradient experiments were performed, and qPCR results showed that CD63 mRNA levels began to increase after 2 h of hypoxia treatment and peaked at 12 h. Similarly, WB analysis indicated that CD63 protein levels exhibited the same dynamic change trend, starting to rise at 2 h and reaching the peak at 12 h, suggesting that its expression is dynamically regulated by hypoxia (Figures 3B–D).

### 3.4 Hypoxia significantly enhances the exosome secretion capacity of tumor cells and promotes lactate enrichment

**FIGURE 4:**
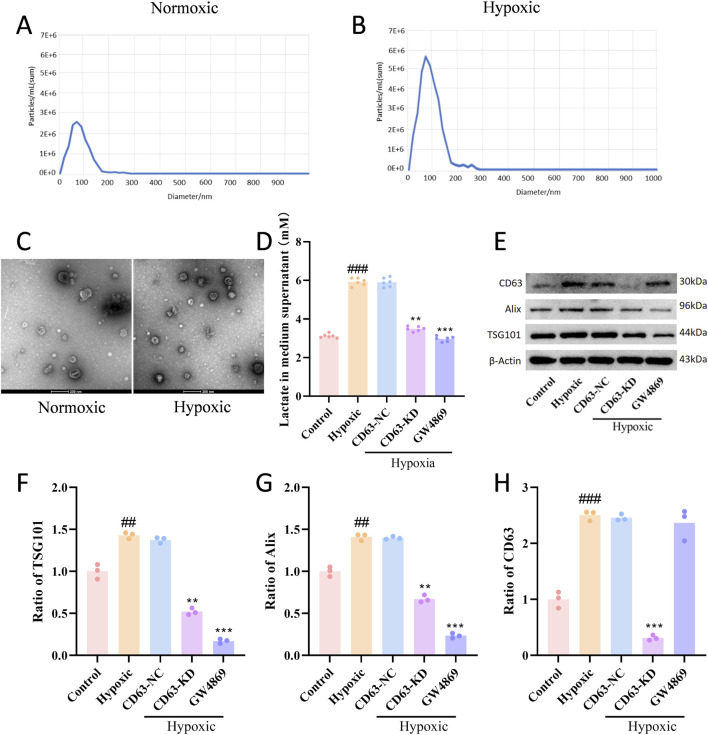
Hypoxia significantly enhances the exosome secretion capacity of tumor cells and promotes lactate enrichment. **(A,B)** The representative NTA particle size distribution graph of exosomes (under normoxic and hypoxic conditions). **(C)** Representative transmission electron microscopy (TEM) images of exosomes isolated from UMT2 cells cultured under normoxic (left) and hypoxic (right) conditions. Scale bar: 200 nm. **(D)** Lactate assay kit detects lactate secretion (n = 6). **(E–H)** The western blots of CD63, AliX and TSG101, as well as the quantitative analysis (n = 3). All data are presented as mean ± SD. The statistical analysis indicated ##*P* < 0.01 and ###*P* < 0.001 in comparison to the control group, while **P < 0.01 and ***P < 0.001 were observed against the Hypoxic group.

Examine the effect of hypoxia on exosome properties derived from tumor cells, we quantitatively analyzed exosomes secreted by UMT2 cells under normoxic and hypoxic conditions using NTA. The results indicated that the concentration of exosome particles in the hypoxic group is much higher than that in the normal group. Both groups displayed a predominant particle size distribution within the 30–150 nm range, which is consistent with the classical characteristics of exosomal size (Figures 4A,B). Furthermore, TEM imaging confirmed the presence of exosomes, and qualitative examination indicated a greater abundance of exosomes in preparations from hypoxic cells, corroborating the NTA data (Figure 4C). Metabolite detection revealed a significant increase in lactate concentration in exosomes from the hypoxic group. When intracellular CD63 expression was downregulated using CD63-specific siRNA, exosomal lactate levels were markedly reduced under hypoxic conditions. Furthermore, treatment with the exosome inhibitor GW4869 (10 μM; an inhibitor of neutral sphingomyelinase) abolished the hypoxia-induced increase in lactate secretion (Figure 4D). Further Western blot analysis of exosomal marker proteins indicated that the expression levels of CD63, Alix, and TSG101 in exosomes derived from the hypoxic group were significantly elevated in comparison to those from the normoxic group, The CD63 protein was nearly undetectable in CD63-KD cells, thereby confirming the essential role of CD63 in hypoxia-induced exosome secretion and lactate loading (Figures 4E–H).

### 3.5 Hypoxic exosomes promote CD63-dependent M2 macrophage polarization and immunosuppressive metabolic reprogramming

**FIGURE 5:**
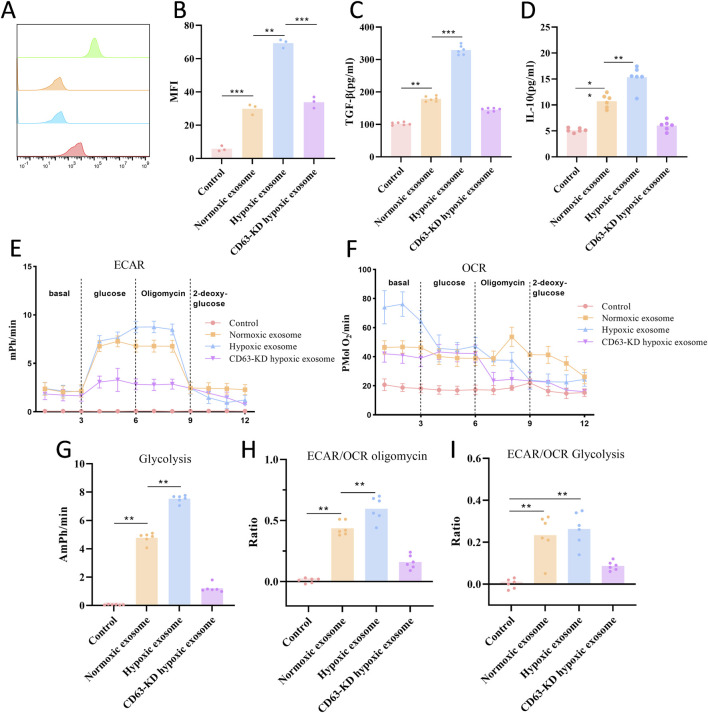
Hypoxic exosomes promote CD63-dependent M2 macrophage polarization and immunosuppressive metabolic reprogramming. **(A,B)** Flow cytometry assessment of CD206 (n = 3). **(C,D)** ELISA method to detect TGF-β and IL-10 immunosuppressive factors (n = 6). **(E,F)** Representative seahorse plots are presented, illustrating the ECAR and OCR during the glycolysis stress test. The dashed lines indicate the time points at which each stimulation commenced. **(G–I)** Quantification of glycolysis, ECAR/OCR during glucose exposure, and ECAR/OCR following oligomycin exposure is provided. **P < 0.01 and ***P < 0.001.

In parallel, hypoxic exosomes significantly induced M2 polarization of ATCC TIB-202 macrophages, as evidenced by an increase in the proportion of CD206^+^ macrophages (Figures 5A,B). This polarization was accompanied by elevated secretion of TGF-β and IL-10 (Figures 5C,D), key cytokines that contribute to the development of immunosuppressive tumor microenvironments. Notably, both effects were markedly attenuated in the CD63-KD exosome group. Metabolic analysis revealed that macrophages treated with hypoxic exosomes exhibited an increase in the ECAR/OCR ratio, indicative of enhanced glycolysis (Figures 5E–I), whereas CD63-KD exosomes mitigated this metabolic shift.

### 3.6 Hypoxic exosomes induce CD63-dependent T cell exhaustion

**FIGURE 6:**
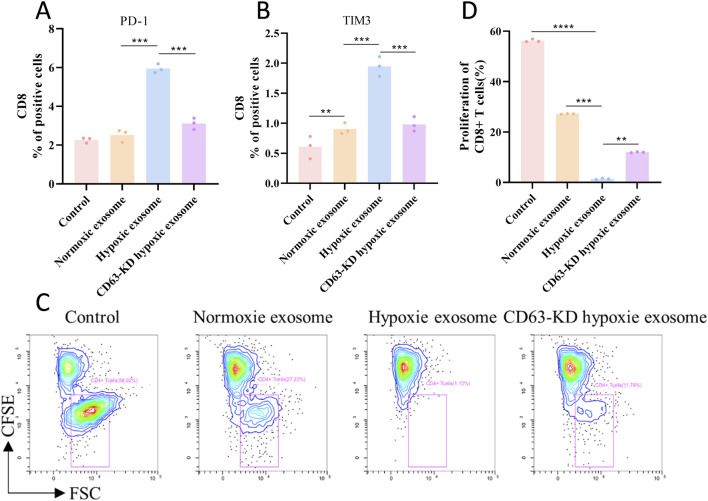
Hypoxic exosomes induce CD63-dependent T cell exhaustion. **(A,B)** Flow cytometry analysis of T cell exhaustion markers (PD-1^+^TIM-3^+^). **(C,D)** T cell killing rate test (n = 3). **P < 0.01, ***P < 0.001 and ****P < 0.0001.

To evaluate the functional impact of hypoxic exosomes on immune cells, human primary CD8^+^ T cells and ATCC TIB-202 macrophages were co-cultured with normoxic and hypoxic tumor-derived exosomes for 48 h. The results exhibited an increase in PD-1^+^TIM-3^+^ double-positive cells, indicative of T cell exhaustion (Figures 6A,B), and demonstrated a decrease in T cell killing (Figures 6C,D), reflecting functional impairment. Notably, these effects were reversed by CD63-knockdown hypoxic exosomes. Collectively, these findings suggest that hypoxic exosomes drive CD63-dependent T cell exhaustion and loss of cytotoxicity, thereby linking CD63 to immune evasion in hypoxic melanoma microenvironments.

## 4 Discussion

This study elucidates a novel mechanism through which hypoxic myeloma cells transport lactate via CD63-positive exosomes, thereby remodeling the immune microenvironment. Existing literature indicates that CD63, a marker of exosomes, demonstrates significantly increased expression under conditions of metabolic stress (Li et al., 2022; Luo et al., 2024). Notably, the elevated expression of CD63 in tumor cells is positively correlated with the risk of lymph node metastasis and poor prognosis in patients with UM (Matsumoto et al., 2025).

CD63, a prominent member of the tetraspanin protein family (Tognoli et al., 2023; Palmulli et al., 2024) is upregulated under hypoxic conditions. This is evidenced by our experimental results: under hypoxic treatment, both the mRNA level of CD63 (detected by qPCR) and its protein level (detected by WB) are significantly increased compared to normoxic conditions. The consistent elevation of CD63 at both transcriptional and translational levels reveals the regulatory effect of hypoxia signaling on CD63 expression. CD63 may enhance exosome biogenesis by stabilizing the ESCRT complex (TSG101, Alix), resulting in a higher concentration of exosome particles in hypoxic groups compared to normoxic groups. The upregulation of TSG101, Alix, and CD63 following hypoxia further supports this finding.

While our data confirm that CD63 knockdown significantly reduces exosomal lactate levels, the precise molecular mechanism by which CD63 facilitates lactate enrichment warrants further investigation. As a key tetraspanin organizing membrane microdomains, CD63 may indirectly promote lactate loading by stabilizing the exosomal membrane and facilitating the recruitment or activity of lactate transporters such as Monocarboxylate Transporters (MCTs). Alternatively, CD63 might influence the sorting of lactate or its regulatory enzymes into exosomes via its interactions with the ESCRT machinery. Future studies are needed to dissect the direct versus indirect roles of CD63 in exosomal metabolite sorting.

The role of exosomal lactate as an intercellular messenger is gaining attention in cancer biology. In other solid tumors, such as hepatocellular carcinoma, exosomal lactate has been shown to promote metastasis by modulating exosome biogenesis (Jiang et al., 2025), and in breast cancer models, exosomes can transfer glycolytic phenotypes associated with chemoresistance (Choudhury et al., 2025). However, within the unique context of uveal melanoma—a tumor characterized by profound intrinsic hypoxia and an immunosuppressive, “cold” microenvironment—the specific mechanisms and consequences of exosomal lactate transfer remain underexplored. Our study not only extends this emerging paradigm to UM but also uniquely identifies the hypoxia-CD63 axis as a pivotal regulator. By linking increased exosomal lactate loading and immune suppression directly to the upregulation of the exosomal tetraspanin CD63 under low oxygen tension, we provide a novel, mechanism-specific insight into how UM may exploit vesicular communication to maintain its immunologically privileged niche.

Lactate, rather than being merely a metabolic byproduct of aerobic glycolysis, serves as a critical signaling molecule and immunoregulator within the TME and in inflammatory contexts. A novel mechanism for its delivery involves exosome-dependent transcellular transport (Thakur et al., 2021; Vahabi et al., 2023; Jiang et al., 2025). Our findings demonstrate that exosomes derived under hypoxic conditions carry significantly elevated levels of lactate compared to those from normoxic conditions. This hypoxic exosomal lactate likely acts as a key mediator that influences immune cell function and drives metabolic phenotype switching. Elevated lactate levels exert immunomodulatory effects through various mechanisms, including metabolic reprogramming, epigenetic modifications, and the activation of specific signaling pathways.

Our observations indicate that macrophages exposed to a hypoxic exosomal environment exhibited a markedly elevated ECAR, indicative of glycolytic dominance, a characteristic metabolic feature associated with M2 polarization (Dai et al., 2025). This metabolic shift is further corroborated by a significant increase in the M2 marker CD206 following hypoxia. In contrast, T cells demonstrated a decreased OCR and impaired mitochondrial oxidative phosphorylation function, which aligns with the observed increase in PD-1^+^TIM-3^+^ exhausted T cells and the overall rate of T-cell exhaustion post-hypoxia. This lactate-induced metabolic reprogramming has profound functional implications: while M1 macrophages exhibit tumor-suppressive capabilities, M2-polarized macrophages (commonly referred to as tumor-associated macrophages) facilitate immunosuppression and promote tumor cell proliferation (Yi et al., 2024; Shi et al., 2025). Similarly, although CD8^+^ T cells are crucial antitumor effectors whose presence is associated with favorable outcomes, the metabolic suppression caused by the lactate-rich TME, evidenced by the shift towards glycolysis (as indicated by a significantly elevated ECAR/OCR ratio) and away from oxidative metabolism, critically undermines their antitumor efficacy (Ping et al., 2024). Therefore, the hypoxia-driven accumulation of lactate within exosomes emerges as a significant mechanism contributing to the immunosuppressive and pro-tumorigenic metabolic reprogramming of key immune cells in the TME.

Although this study primarily focuses on UM, the Hypoxia/CD63/exosomal lactate axis may also be pertinent to other solid tumors characterized by hypoxic microenvironments. Targeting CD63 or lactate transporters, such as monocarboxylate transporter 1, could disrupt this immunosuppressive circuit, potentially enhancing responses to immunotherapy.

Building on our findings, several concrete therapeutic strategies could be envisioned to disrupt the hypoxia/CD63/exosomal lactate axis. These include: (1) developing CD63-blocking antibodies or small-molecule inhibitors to interfere with exosome biogenesis or uptake; (2) employing inhibitors of exosome biogenesis or secretion, such as molecules targeting neutral sphingomyelinase 2 (nSMase2) or key Rab GTPases; and (3) using pharmacological inhibitors of lactate transporters, primarily monocarboxylate transporters 1 and 4 (MCT1/4), to impede lactate efflux from tumor cells or its uptake by immune cells. However, translating these strategies faces significant challenges. Achieving sufficient tumor specificity to minimize systemic toxicity is paramount. The potential for compensatory mechanisms within the robust tumor metabolic network also necessitates careful consideration, possibly favoring combination approaches. Furthermore, developing suitable delivery systems (e.g., nanoparticles, targeting ligands) will be crucial to enhance drug bioavailability and reduce off-target effects. Future research should focus on validating these interventions in preclinical models and exploring their synergy with existing immunotherapies.

The molecular heterogeneity of uveal melanoma should be considered when interpreting our findings. This study primarily utilized the GNA11-mutant UMT2 cell line. UM, however, encompasses other key molecular subtypes characterized by mutually exclusive mutations, such as in GNAQ, as well as alterations in BAP1 which are associated with higher metastatic risk. While the hypoxia/CD63/exosomal lactate axis identified here presents a novel immunosuppressive mechanism, its operation and relative importance may vary across these different genetic contexts. Future studies employing models of distinct molecular subtypes and primary patient-derived cells are necessary to fully elucidate the generalizability and potential adaptations of this pathway within the broader UM landscape.

The molecular heterogeneity of uveal melanoma should be considered when interpreting our findings. This study primarily utilized the GNA11-mutant UMT2 cell line. UM, however, encompasses other key molecular subtypes characterized by mutually exclusive mutations, such as in GNAQ, as well as alterations in BAP1 which are associated with higher metastatic risk. While the hypoxia/CD63/exosomal lactate axis identified here presents a novel immunosuppressive mechanism, its operation and relative importance may vary across these different genetic contexts. Future studies employing models of distinct molecular subtypes and primary patient-derived cells are necessary to fully elucidate the generalizability and potential adaptations of this pathway within the broader UM landscape.

The limitations of this study include: (1) The exclusive use of immortalized cell lines, which necessitates validation with primary UM cells; (2) The absence of metabolomic analysis of patient-matched exosome samples; (3) Lack of *in vivo* experiments. (4) There are no interventions to inhibit lactic acid transport. Developing bifunctional molecules that simultaneously inhibit CD63 exosome release and lactate transport may help overcome the compensatory mechanisms associated with single-target approaches, thereby representing a novel avenue for combination therapies.

## 5 Conclusion

We demonstrate that CD63-mediated exosomal lactate shuttling under hypoxia drives immunosuppression in UM by inducing M2 macrophage polarization and T cell exhaustion. This mechanism highlights the hypoxia/CD63/exosomal lactate axis as a promising therapeutic target to disrupt the immunosuppressive tumor microenvironment and potentially sensitize UM to immunotherapy.

## Data Availability

The single-cell RNA-seq dataset used in this study was downloaded from the GEO database (GSE139829).
